# Overexpression of a maize plasma membrane intrinsic protein ZmPIP1;1 confers drought and salt tolerance in *Arabidopsis*

**DOI:** 10.1371/journal.pone.0198639

**Published:** 2018-06-01

**Authors:** Lian Zhou, Jing Zhou, Yuhan Xiong, Chaoxian Liu, Jiuguang Wang, Guoqiang Wang, Yilin Cai

**Affiliations:** Maize Research Institute, Key Laboratory of Biotechnology and Crop Quality Improvement, College of Agronomy and Biotechnology, Southwest University, Chongqing, China; National Taiwan University, TAIWAN

## Abstract

Drought and salt stress are major abiotic stress that inhibit plants growth and development, here we report a plasma membrane intrinsic protein ZmPIP1;1 from maize and identified its function in drought and salt tolerance in *Arabidopsis*. ZmPIP1;1 was localized to the plasma membrane and endoplasmic reticulum in maize protoplasts. Treatment with PEG or NaCl resulted in induced expression of *ZmPIP1;1* in root and leaves. Constitutive overexpression of *ZmPIP1;1* in transgenic *Arabidopsis* plants resulted in enhanced drought and salt stress tolerance compared to wild type. A number of stress responsive genes involved in cellular osmoprotection in *ZmPIP1;1* overexpression plants were up-regulated under drought or salt condition. *ZmPIP1;1* overexpression plants showed higher activities of reactive oxygen species (ROS) scavenging enzymes such as catalase and superoxide dismutase, lower contents of stress-induced ROS such as superoxide, hydrogen peroxide and malondialdehyde, and higher levels of proline under drought and salt stress than did wild type. *ZmPIP1;1* may play a role in drought and salt stress tolerance by inducing of stress responsive genes and increasing of ROS scavenging enzymes activities, and could provide a valuable gene for further plant breeding.

## Introduction

Plants live in constantly changing environments that are often stressful for growth and development. These adverse environmental conditions include biotic stress and abiotic stress. Drought and salt stress are major abiotic stress that limit plant productivity in agriculture, and threaten food security [1]. The adaptive responses of plants to the stress can be conceptually grouped into three aspects: osmotic adjustment or ion homeostasis caused by drought and salt; stress damage control and repair, detoxification; and growth control [2]. Besides osmotic stress caused by drought and salt, oxidative damage is another stress resulting from imbalance between reactive oxygen species (ROS) production and scavenging [3]. The ROS scavenging mechanisms have been proven in the response of plants to water deficit and salinity functioning as toxic by-products of stress metabolism, as well as important signal transduction molecules [4].

Water is the most important constituent of any living cell. Water uptake from the soil to the root and transport in the plant is vital to many physiological processes of plants. There are three different pathways of water transport through plant tissues: the apoplastic path, the symplastic path and the transmembrane path [5]. The later two path together form the cell-to-cell path, in which water moves through plasmodesmata or across membranes [6]. Apoplastic water flow can be blocked by Casparian bands and suberin lamellae [7], thus the cell-to-cell path is essential to water transport across membranes. The transcellular water movement is tightly controlles by the activity and amount of aquaporins (AQPs) [5]. Aquaporins are suggested to play critical roles in plant water transport and water balance [8–12].

Aquaporins belong to major intrinsic protein (MIP) family. MIP homologs show a highly conserved structures wherein six membrane-spanning alpha helices are linked by five loops with their N- and C-termini into the cytosol. Two of these loops contain highly conserved asparagine- proline- alanine (NPA) motifs, which are of importance in the formation of water-selective channel [13]. In higher plants, AQPs are categorized into five subfamilies, the plasma membrane intrinsic proteins (PIPs), the tonoplast intrinsic proteins (TIPs), and the nodulin26-like intrinsic proteins (NIPs), the small basic intrinsic proteins (SIPs) and the uncategorized intrinsic proteins (XIPs) [14]. The PIPs are considered as the main water transport pathway across plasma membranes in root and leaf tissues that play important roles in plant water relations [11,12,14,15]. Although PIPs are conserved throughout all higher plants, they are further divided into PIP1s and PIP2s according to the N terminal length of the proteins. PIP1s and PIP2s may interact either within the membrane to co-expression and show high water permeability in *Xenopus laevis* oocytes [16–22].

Aquaporin genes have been characterized in several plant species, such as *Arabidopsis thaliana* [23], *Oryza sativa* [24], *Zea mays* [13], soybean [25] and tomato [26]. The high diversity in plant aquaporins suggests variation of their physiological roles. Abiotic stress such as salt, drought and cold influence the water balance of plants and the expression of aquaporin genes [27]. Studies with overexpression of several aquaporin genes in different plants confer water-deficit stress tolerance. Overexpressing *NtAQP1* in tobacco increased photosynthetic rate, water use efficiency and yield under salt stress [28]. Overexpression of PIP genes in rice, soybean and banana increased salt, drought or cold tolerance of the transgenic plants, and increased yields in the field [29–31]. All the reports implying the complexity of PIPs function in plants.

Maize (*Zea mays* L.) as an important food, feed and industrial crop, is a major crop in the world’s agriculture production. Drought and salt has been the severe environmental stress affecting maize production, developing maize with drought and salt tolerance continues to be an important objective of increasing maize production. Although several *ZmPIP* genes have been identified in maize, but little is known about the function of *ZmPIP* genes, especially the *ZmPIP* genes that involve in abiotic stress tolerance have not been functional characterized.

According to biological phenotype of transgenic materials in response to drought and salt stress, the function of ZmPIP1;1 will be analyzed. The study may set a foundation for the underlying mechanism of *ZmPIP1;1* gene on plant drought and salt tolerance, and to provide useful gene resources for maize breeding project seeking to improve abiotic stress tolerance.

## Materials and methods

### Plant materials, growth conditions

The maize inbred line B73 was used for physiological experiments. The root, stem, leaf, immature tassel (3–4 cm), ear (3–4 cm), endosperm and embryo 18 day after pollination (DAP) were collected for total RNA extraction. All tissues were immediately frozen in liquid nitrogen and subsequently stored at -80°C. Plants were grown in green house under 12 h light/12 h dark photoperiod with light intensity of 1000 μmol m^-2^ sec^-1^ and day/night temperatures of 30/22°C. Humidity of the growth room was controlled at approximately 30%.

*Arabidopsis thaliana* (Col-0) seeds were surface-sterilized and sown on MS plates, and stratified for three days at 4°C. *Arabidopsis thaliana* plants were grown in an environmental controlled growth chamber programmed for a 16 h light/8 h dark photoperiod with daytime temperature of 24°C and a night temperature of 21°C.

### Stress treatments

For stress treatments, B73 seeds were germinated in nursery pots with soil. Five days after germination, the seedlings grown uniformly were transferred into pots with nutrient solution. 1/2 Hoagland solution was used for hydroponic culture containing 6.0 mM KNO_3_, 4.0 mM Ca(NO_3_)_2_, 1.0 mM KH_2_PO_4_, 0.05 mM KCl, 1.0 mM MgSO_4_, 0.02 mM Fe-EDTA(Na), 25 μM H_3_BO_3_, 0.2 μM MnSO_4_, 0.2 μM ZnSO_4_, 0.05 μM (NH_4_)_2_MoO_4_, 0.05 μM CuSO_4_. Seven-day-old maize seedlings were transferred into Hoagland solutions supplied with 10% PEG6000 and 100 mM NaCl, growth chamber for indicated periods, respectively. Three independent experiments were designed for each treatment.

*Arabidopsis thaliana* (Col-0) plants grown on MS agar plates (0.8% agar) were used in stress treatment experiments. Five-day-old *Arabidopsis* seedlings were transplanted into MS medium plates with 150 mM Mannitol or 100 mM NaCl. For drought treatment in soil culture, watering was withheld from 2-week-old plants for 15 days. Then plants were re-watered for 1 week before the photograph was taken. For salt treatment in soil culture, 2-week-old plants were subjected to salt stress by watering with 100 mM NaCl for 15 days at 3 days intervals.

### Water loss analysis

Rosette leaves from 2 week old seedlings were detached, weighed and placed on paper under the normal condition for *Arabidopsis* growth. Fresh weight of rosette leaves was measured each hour for 5 hours. Water loss percentage was calculated as the loss in fresh weight of samples.

### Subcellular localization

Full length cDNA of *ZmPIP1;1* without stop code was amplified via PCR using the primers in S1 Table. The PCR product was cloned into vector pCAMBIA1302 under the control of the CaMV 35S promoter. The resulting construct (pCAMBIA1302: *ZmPIP1;1*) placed *ZmPIP1;1* in-frame, upstream of the sGFP. Maize mesophyll protoplast isolation and polyethylene glycol-mediated transformation were modified as previously [32]. In brief, 10 μg plasmid DNA of each construct was transformed into 0.2 mL protoplast suspension. The plasma membrane marker (pm-rk; The Arabidopsis Information Resource stock no. CD3-1007) and endoplasmic reticulum marker (ER-rk; The Arabidopsis Information Resource stock no. CD3-959) were described by Nelson [33]. After incubation at 30°C dark for 12 to 15 h, fluorescence signals in maize protoplasts were detected. Confocal microscopy images were taken using a LSM700 confocal laser scanning microscope (Carl Zeiss). Excitation/emission wavelengths were 488 nm/506 to 538 nm for YFP and GFP, and 561 nm/575 to 630 nm for mCherry.

### Construction of transgenic plants

Full-length cDNA of *ZmPIP1;1* was amplified by PCR with cDNA of B73 and ligated into pMD-18 T vector (Takara). After sequencing, the correct *ZmPIP1;1* was digested from pMD-18 T vector using *Bam*HI and *Xba*I restriction enzymes. *ZmPIP1;1* was then cloned into binary plasmid pCambia3300 which was modified by introducing CaMV 35S promoter and nos terminator. The vector was transformed into *Arabidopsis thaliana* ecotype Col-0 using the floral dip method mediated by *Agrobacterium tumefaciens* strain EHA101. Transgenic plants were screened with Glufosinate (5 mg/L, Sigma) and confirmed by PCR analysis.

### RNA extraction

Total RNA was isolated from tissues of Maize B73 using RNAprep pure Plant Kit (TIANGEN) according the manufacturer’s instruction. 50 mg maize tissues with three biological replicate were quickly harvested, frozen in liquid nitrogen and stored at -80°C. Total RNA was quantified with nanodrop (Thermo).

### Quantitative RT-PCR

First-strand cDNAs were synthesized from total RNA using RevertAid First Strand cDNA Synthesis Kit (Thermo Scientific). qRT-PCR was performed using a SYBR Green (TOYOBO, OSAKA) on a CFX96 Touch™ Cycler (Bio-Rad) system, according to the manufacturer’s instructions. The amplification program for SYBR Green I was performed at 94°C for 10 sec, 58°C for 10 sec and 72°C for 10 sec. Triplicate quantitative assays were performed on each cDNA sample. The relative level of expression was calculated using the formula 2 ^-△(△cp)^. The housekeeping gene *ZmACT3*, *AtACT2* and *AtUBQ10* was used as an internal control. All primers used for qRT-PCR are given in S1 Table.

### Expression of stress responsive genes in transgenic *Arabidopsis*

Expression of stress responsive genes in 14-day-old *ZmPIP1;1*-Oe transgenic plants and WT seedlings dehydrated on paper at 24°C and 60% humidity under normal light for 2 hours for drought treatment or watering with 100 mM NaCl for 6 hours for salt treatment. The expression of genes *RD29A*, *RD29B*, *LEA18*, *TRX5*, *P5CS1* and *P5CS2* involved in specific stress signaling pathways in *ZmPIP1;1* transgenic *Arabidopsis* plants was analyzed by qRT-PCR to study the mechanism of *ZmPIP1;1* mediated drought and salt tolerance and the gene’s role in the drought and salt tolerance pathway. The primers are listed in S1 Table.

### Determining proline and malondialdehyde content

For proline and malondialdehyde content analysis, 28-day-old plants were watering without or with 100mM NaCl for control or salt treatment for 7 days at 3 day intervals, and no watering for drought treatment, and then harvested for analysis. Leaves and root from *ZmPIP1;1*-Oe transgenic plants and WT were sampled and 50 mg of the fresh material was weighed. Proline content determination was performed as reported previously [34]. Malondialdehyde content was determined by the thiobarbituric acid (TBA)-based colorimetric method as described [35].

### ROS analysis and antioxidant enzyme activity assay

For ROS analysis and antioxidant enzyme activity assay, 28-day-old plants were watering without or with 100mM NaCl for control or salt treatment for 7 days at 3 day intervals, and no watering for drought treatment, and then harvested for analysis. Superoxide and hydrogen peroxide were analyzed as described [36,37]. For extraction of catalase and superoxide dismutase, about 0.5 g of samples was ground in liquid and homogenized in 5 ml of extraction buffer containing 0.05 M phosphate buffer (pH 7.8) and 1% polyvinyl pyrrolidone. The homogenate was centrifuged at 10,000 g for 10 min at 4°C and the supernatant was collected for enzyme activity analysis. The activities of catalase and superoxide dismutase were spectrophotometrically measured as modified [38].

## Results

### Subcellular localization of ZmPIP1;1

**Fig 1:**
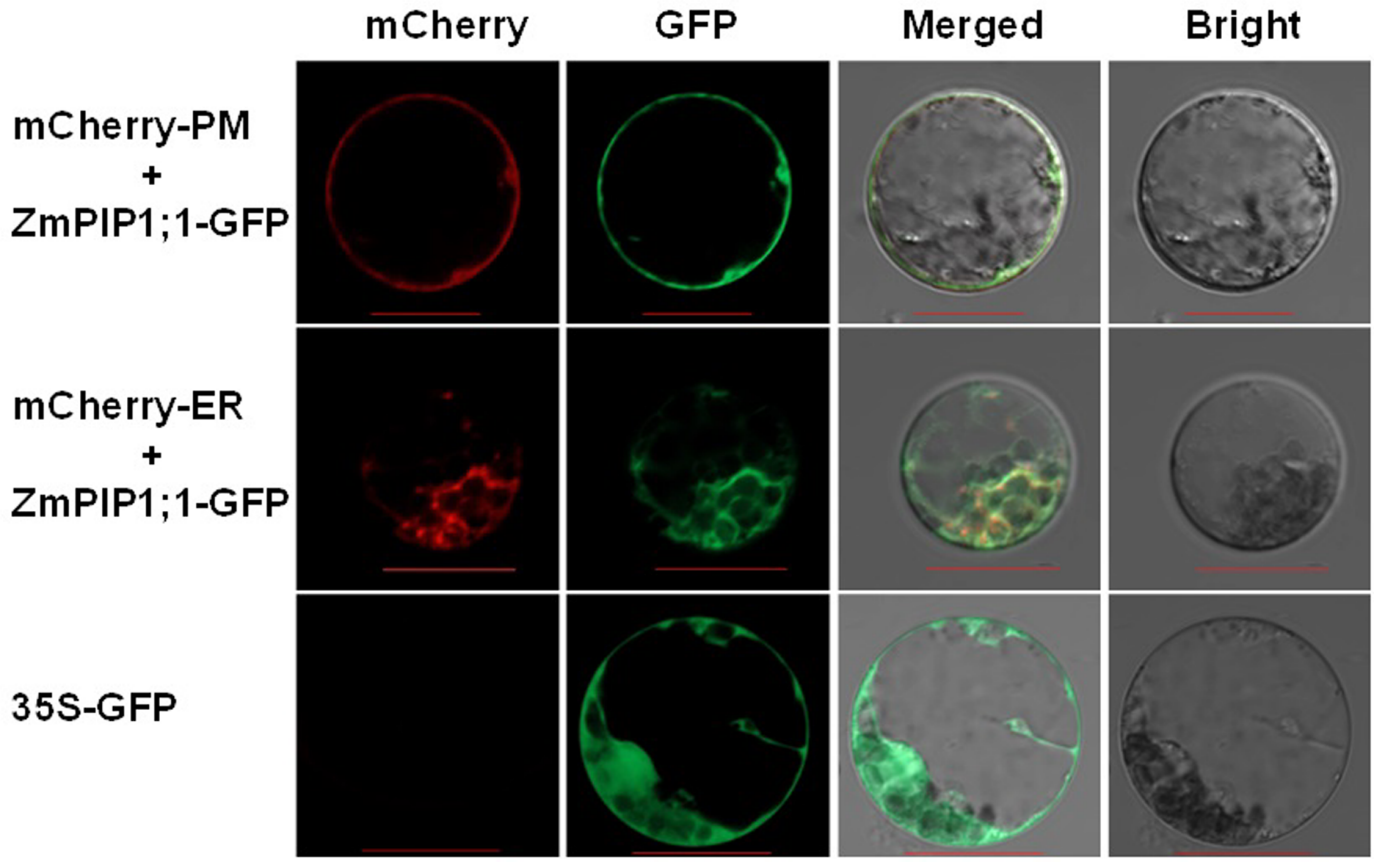
Subcellular localization of ZmPIP1;1 in maize protoplasts. ZmPIP1;1-GFP was colocalized with a mCherry-labeled plasma membrane marker (pm-rk; CD3-1007) and a mCherry-labeled endoplasmic reticulum marker (ER-rk; CD3-959), respectively. The vector pCAMBIA1302 that sGFP was under the control of the CaMV 35S promoter served as control. Bars = 20 μm.

The full-length CDS of *ZmPIP1;1* (Accession No. X82633) was amplified by PCR using cDNA isolated from the leaves of maize B73 seedlings. The cDNA of *ZmPIP1;1* is comprised of 1169 bp with an 864 bp open reading frame. The *ZmPIP1;1* shares the same conserved structural domains as the AQPs and belongs to the PIP1subgroup. A phylogenetic tree was constructed based on the amino acid sequence alignment of ZmPIPs and other plant species AQPs sequences obtained from GenBank (S1 Fig). Phylogenetic analysis showed that ZmPIP1;1 had a high degree of sequence similarity with other AQPs. ZmPIP1;1 protein contains the characteristic motifs of PIPs and is predicted to be located on the plasma membrane. To verify the subcellular localization of ZmPIP1;1, the full length of ZmPIP1;1 protein without stop code was fused with green fluorescent protein (GFP) and driven by a constitutive Cauliflower mosaic virus (CaMV) 35S promoter. The subcellular localization of ZmPIP1;1 was assessed using maize protoplasts isolated from leaves of maize seedlings. We conducted co-localization of ZmPIP1;1-GFP with a plasma membrane marker (pm-rk; CD3-1007) and an endoplasmic reticulum marker (ER-rk; CD3-959), respectively. ZmPIP1;1distributed on the external membrane and inner network of the protoplasts, and overlapped with the red fluorescence of mCherry. The results indicated that ZmPIP1;1 was localized on the plasma membrane and endoplasmic reticulum (Fig 1).

### Induced expression of *ZmPIP1;1* under stress conditions

**Fig 2:**
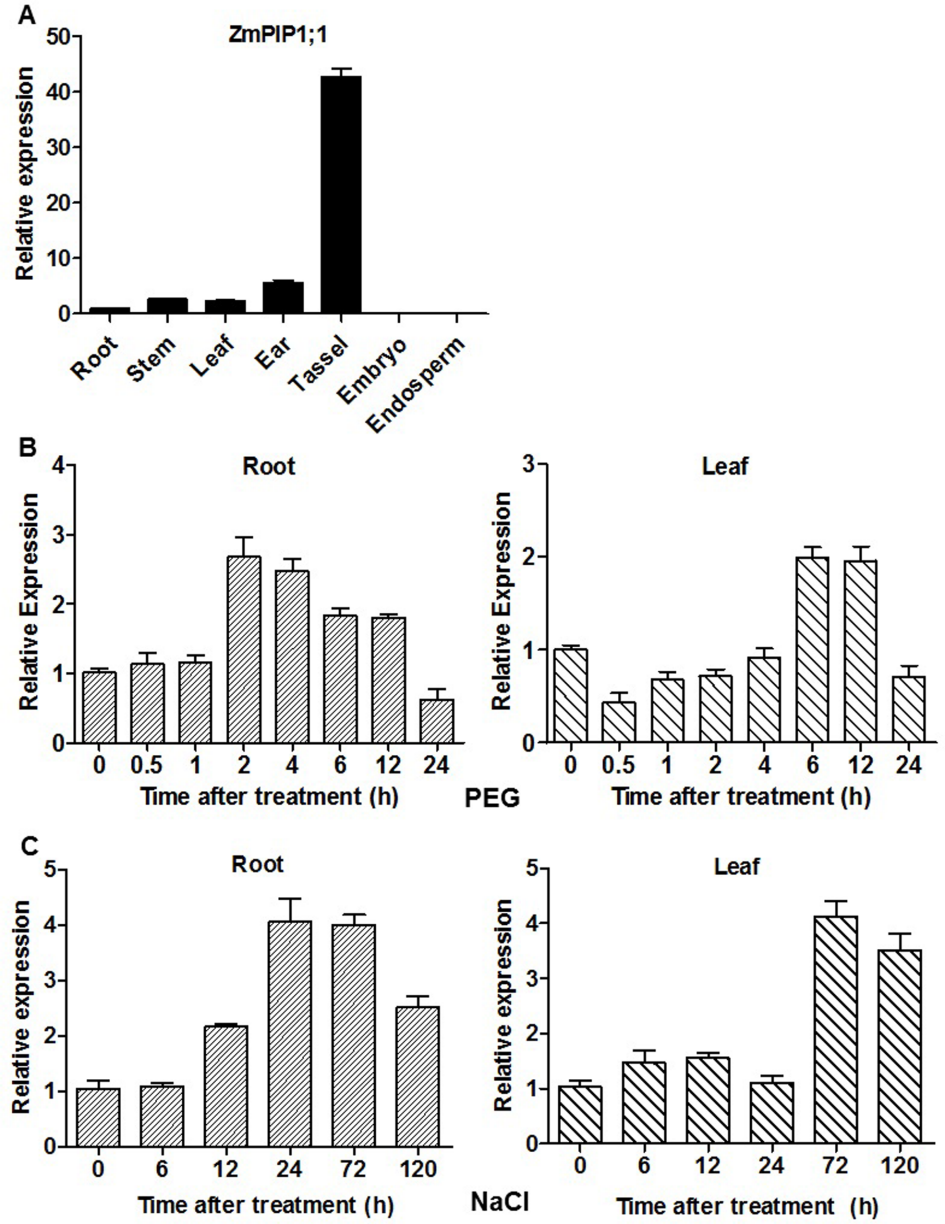
Expression pattern of *ZmPIP1;1* under normal and stress conditions. (A) Relative expression levels of *ZmPIP1;1* in root, stem, mature leaf, immature tassel (3–4 cm), immature ear (3–4 cm), 18 DAP endosperm and embryo. Maize seedlings were grown in nutrient solution. Total RNA was extracted from different tissues for qRT-PCR. (B) Trifoliolate maize seedlings were treated with or without 10% PEG in nutrient solution. (C) Trifoliolate maize seedlings were treated with or without 100 mM NaCl in nutrient solution. RNA was extracted from the root and leaf of these seedlings at different time after treatment. All data are means of three biological replicates with error bars indicating SD. Expression level of treated plants was relative to control plants at each time point.

To analyze *ZmPIP1;1* gene expression in different maize tissues, we measured the expression of *ZmPIP1;1* in root, stem, mature leaf, immature tassel (3–4 cm), immature ear (3–4 cm), 18 DAP endosperm and embryo were isolated from inbred line B73 by qRT-PCR. This result showed that *ZmPIP1;1* was highly expressed in tassel, ear, leaf, stem and root whereas it was lowly expressed in embryo and endosperm (Fig 2A). To characterize the response of *ZmPIP1;1* to osmotic stress, we determined expression of *ZmPIP1;1* in root and leaf after 10% PEG treatment for 0.5, 1, 2, 4, 6, 12 and 24 hours. The expression of *ZmPIP1;1* was induced by PEG treatment in 2 hours and persistently expression high until 12 hours in root. However, expression was suppressed by PEG treatment in 0.5 hour in leaves. Then expression was induced in the leaves in 6 and 12 hours (Fig 2B). To investigate the response of *ZmPIP1;1* to salt stress, we measured expression of *ZmPIP1;1* in root and leaf after 100 mM NaCl treatment for 6, 12, 24, 72 and 120 hours. The expression of *ZmPIP1;1* was induced by NaCl treatment in 12 hours and further increased at 24 and 72 hours of NaCl treatment in root. However, expression was induced by NaCl treatment in 72 and 120 hours in leaves (Fig 2C).

### Enhanced stress tolerance in *ZmPIP1;1* overexpression *Arabidopsis*

**Fig 3:**
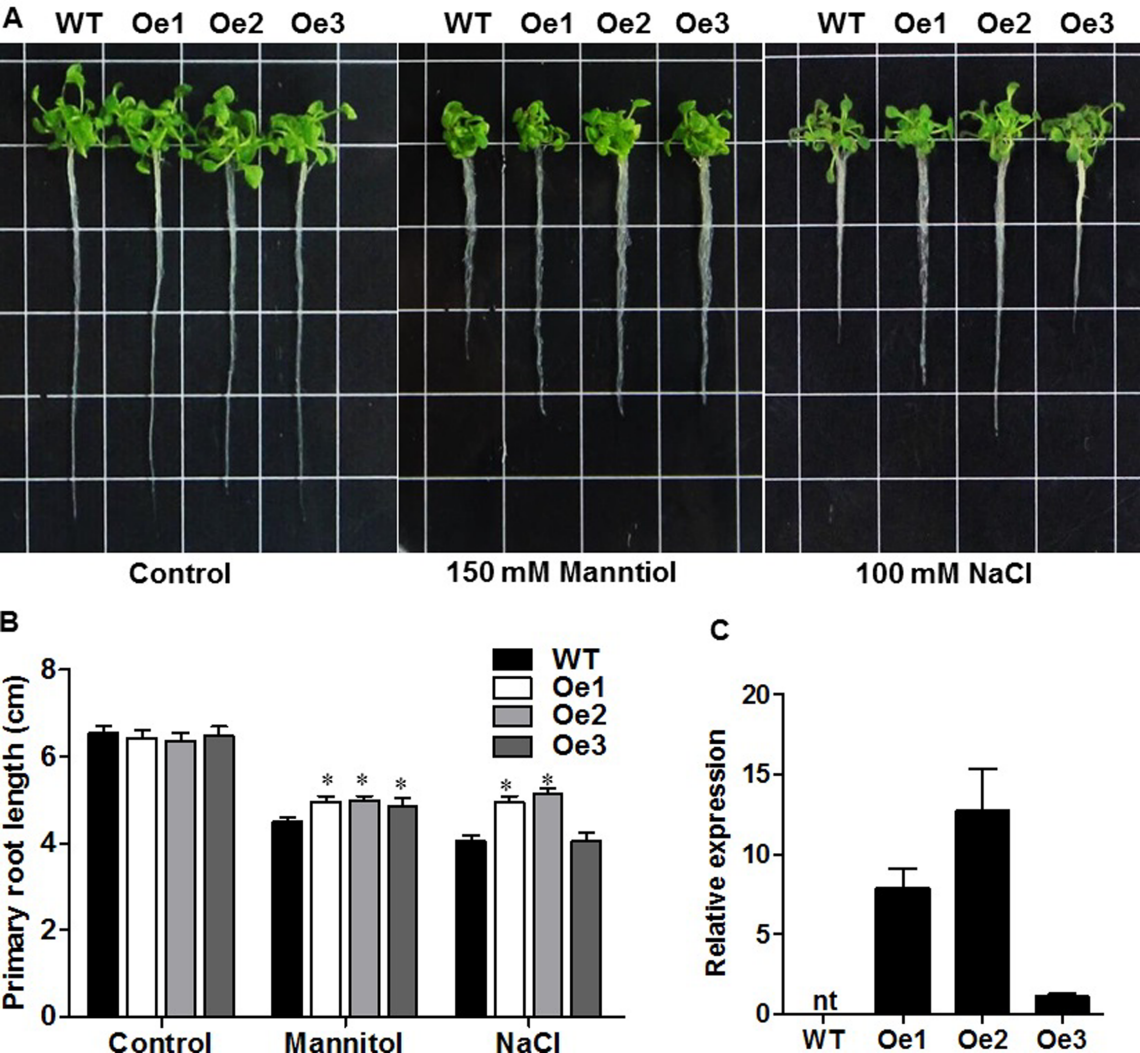
Drought and salt stress tolerance of *ZmPIP1;1* overexpression transgenic lines. Five-day-old WT and *ZmPIP1;1*-Oe transgenic *Arabidopsis* seedlings were treated with 150 mM Manntiol or 100 mM NaCl in MS medium for 7 days. (A) Photographs of WT and *ZmPIP1;1*-Oe transgenic *Arabidopsis* seedlings with 150 mM Manntiol or 100 mM NaCl in MS medium for 7 days. Bar = 1 cm. (B) Primary root length in WT and *ZmPIP1;1*-Oe with 150 mM Manntiol or 100 mM NaCl in MS medium for 7 days. All data are means of 15 biological replicates with error bars indicating SD, *P<0.01. (C) qRT-PCR analysis of three representative *ZmPIP1;1* overexpression transgenic lines. RNA was extracted from the leaves of fourteen-day-old seedlings. All data are means of three biological replicates with error bars indicating SD. Expression of *AtACT2* was used as the internal control.

To investigate the biological functions of *ZmPIP1;1*, we generated transgenic *Arabidopsis* plants that overexpressed *ZmPIP1;1* under the control of CaMV 35S promoter (S3 Fig). Three overexpression transgenic lines (*ZmPIP1;1* -Oe) were selected for further analysis based on the expression of *ZmPIP1;1* (Oe1, Oe2, Oe3) (Fig 3C). Five-day-old wild type (WT, Col-0) and *ZmPIP1;1*-Oe seedlings were tested for tolerance to osmotic and salt stress. It was evident that *ZmPIP1;1*-Oe had less inhibition on root growth on the medium containing 150 mM mannitol or 100 mM NaCl for 7 days, compared with WT, but showed no significant difference in shoot growth with WT (Fig 3A). The corresponding primary root length was consistent with the phenotypes (Fig 3B). These indicated that the *ZmPIP1;1*-Oe was more tolerant to osmotic and salt stress than WT plants.

**Fig 4:**
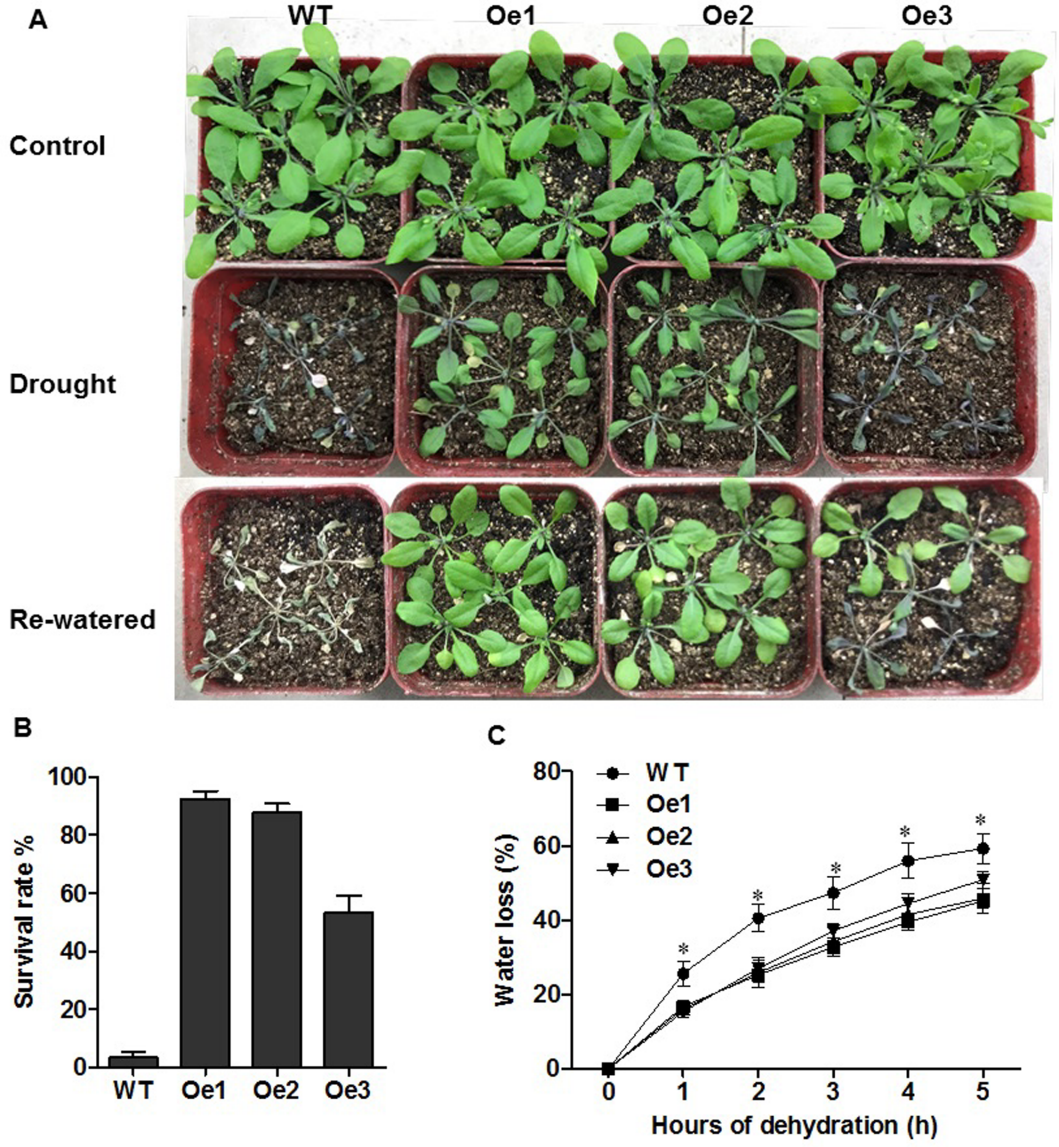
Phenotypes of *ZmPIP1;1* overexpression transgenic lines under drought treatment. (A) Growth of WT and *ZmPIP1;1*-Oe transgenic *Arabidopsis* seedlings under normal and dehydration conditions for 15 days. The re-watered photographs were taken after the plants were re-watered for another 7 days. (B) Survival rates of indicated genotypes upon re-watering for 1 week after dehydration treatment. Three independent repeats were performed, each data are means of 30 plants with error bars indicating SD. (C) Water loss from 0.5 g of detached leaves, from WT and *ZmPIP1;1*-Oe transgenic line, was measured at different times. Three independent repeats were performed, each data are means of 15 plants with error bars indicating SD, *P<0.01.

We further tested the growth of WT and *ZmPIP1;1*-Oe plants, under drought treatment in soil culture. WT and *ZmPIP1;1*-Oe plants were grown in normal condition for two weeks and then subjected to drought condition. There was no difference between WT and *ZmPIP1;1*-Oe plants in normal condition (Fig 4A). After 15 days of water withdrawal, *ZmPIP1;1*-Oe plants did not wilt as severely as WT. And after re-watered and cultured for one week, *ZmPIP1;1*-Oe plants resumed grow, but WT were not able to recover (Fig 4A).The *ZmPIP1;1*-Oe plants showed a significantly higher survival rate than WT (Fig 4B). This might result from a more rapid loss of water in WT plants than in *ZmPIP1;1*-Oe plants (Fig 4C). We measured the loss of water in WT and *ZmPIP1;1*-Oe plants, and result showed that the leaves of WT plants lost about 60% of their original fresh weight, whereas leaves of *ZmPIP1;1*-Oe plants lost only 45–50% from their original fresh weight (Fig 4C). Also, we tested the growth of WT and *ZmPIP1;1*-Oe plants under salt treatment in soil culture. After 15 days watering with 100 mM NaCl, both WT and *ZmPIP1;1*-Oe plants showed chlorosis, and the shoot fresh weight of *ZmPIP1;1*-Oe plants were significantly higher compare to WT (S4 Fig). All these results suggested that overexpression of *ZmPIP1;1* improved plant tolerance to drought and salt stress.

### Induced expression of stress responsive genes in overexpression plants under stress conditions

**Fig 5:**
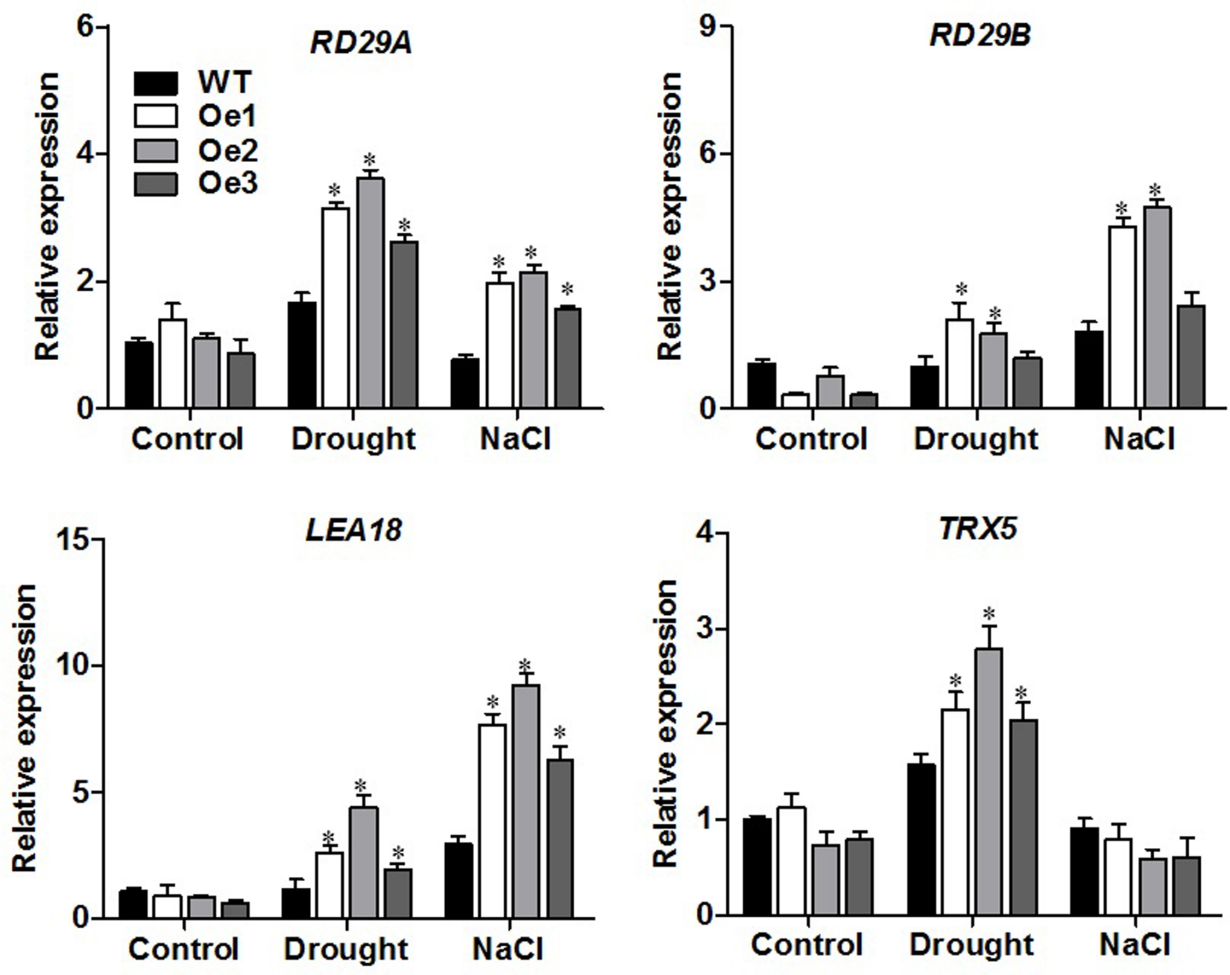
Expression analysis of *Arabidopsis* osmotic stress responsive genes. The expression of *RD29A*, *RD29B*, *LEA18* and *TRX5* under normal, drought or salt stress conditions. RNA was extracted from shoots of two-week-old WT and *ZmPIP1;1* overexpression seedlings under normal condition dehydrated for 2 hours for drought treatment or watering with 100 mM NaCl for 6 hours for salt treatment. All data are means of three biological replicates with error bars indicating SD, *P<0.01. Expression of *AtUBQ10* was used as the internal control.

To evaluate the implications of *ZmPIP1;1* gene in stress response pathways, the expression of genes involved in certain stress signaling pathways in *Arabidopsis* plants was analyzed by qRT-PCR (Fig 5). The expression of the ABA-induced gene *RD29A* and *RD29B* [39] had the most significant increased in *ZmPIP1;1*-Oe transgenic plants, being higher than in the WT plants under drought and salt treatment. *LEA18* gene that function in osmotic regulation and/or protection of cellular structure under dehydration conditions [40] were significantly enhanced in *ZmPIP1;1*-Oe transgenic plants under drought and salt treatment. The expression of an oxidative-induced gene, *TRX5* [41], was significantly induced in *ZmPIP1;1*-Oe transgenic plants than in the WT plants under drought stress but not under salt stress. These results might suggest that overexpression of *ZmPIP1;1* up-regulated the expression of stress responsive genes under the drought and salt condition and also imply that *ZmPIP1;1* played a regulator role in the osmotic stress response signaling pathway.

### Decreased ROS and increased activities of ROS scavenging enzymes in overexpression plants under stress conditions

**Fig 6:**
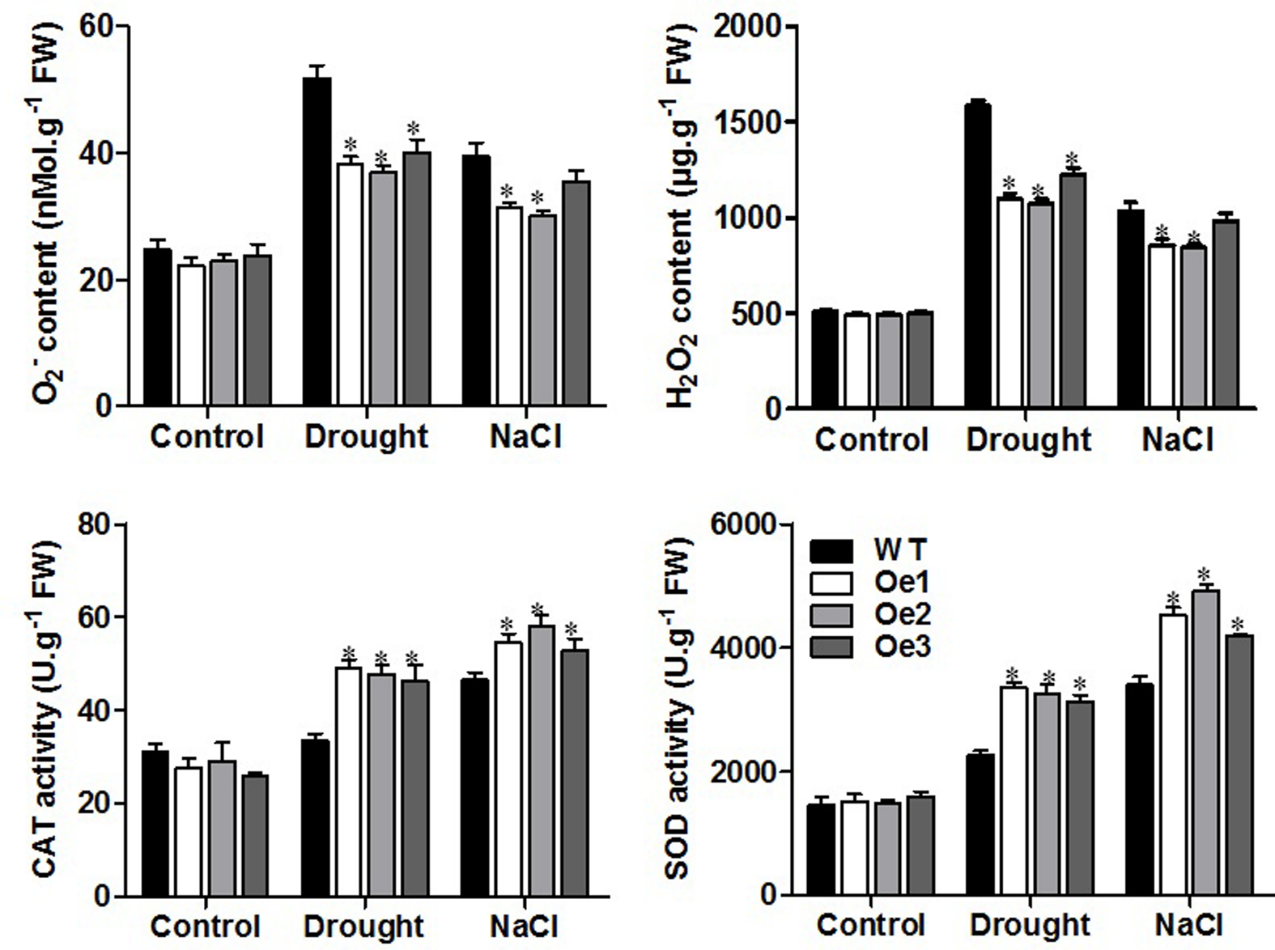
Contents of superoxide and hydrogen peroxide and activities of reactive oxygen species scavenging enzymes in WT and transgenic *Arabidopsis* plants with drought or salt stress. Data are means of four biological replicates with error bars indicating SD. Asterisks indicate a significant difference between the WT and the transgenic lines (*P<0.01).

ROS are generated in plants under oxidative stress, to estimate the content of ROS such as superoxide (O_2_^-^) and hydrogen peroxide (H_2_O_2_) and activities of antioxidant enzymes such as catalase (CAT) and superoxide dismutase (SOD), drought or salt stress treatment were taken. WT and *ZmPIP1;1*-Oe transgenic plants were grown on soil for 28 days and regularly watered without or with 100mM NaCl for control or salt treatment, and no watering for drought treatment. In normal growth conditions, contents of O_2_^-^ and H_2_O_2_ and activities of CAT and SOD displayed no difference between transgenic lines and the WT (Fig 6). After watered with 100mM NaCl for salt treatment, and no watering for drought treatment, *ZmPIP1;1*-Oe transgenic plants had significantly lower O_2_^-^ and H_2_O_2_ contents than the WT, and CAT and SOD activities were significantly higher in *ZmPIP1;1*-Oe transgenic plants under drought or salt stress (Fig 6). These results indicated that reduction of oxidative stress in *ZmPIP1;1*-Oe transgenic plants under drought or salt stress was likely related to increased activities of ROS scavenging enzymes.

### Increased levels of malondialdehyde and proline in overexpression plants under stress conditions

**Fig 7:**
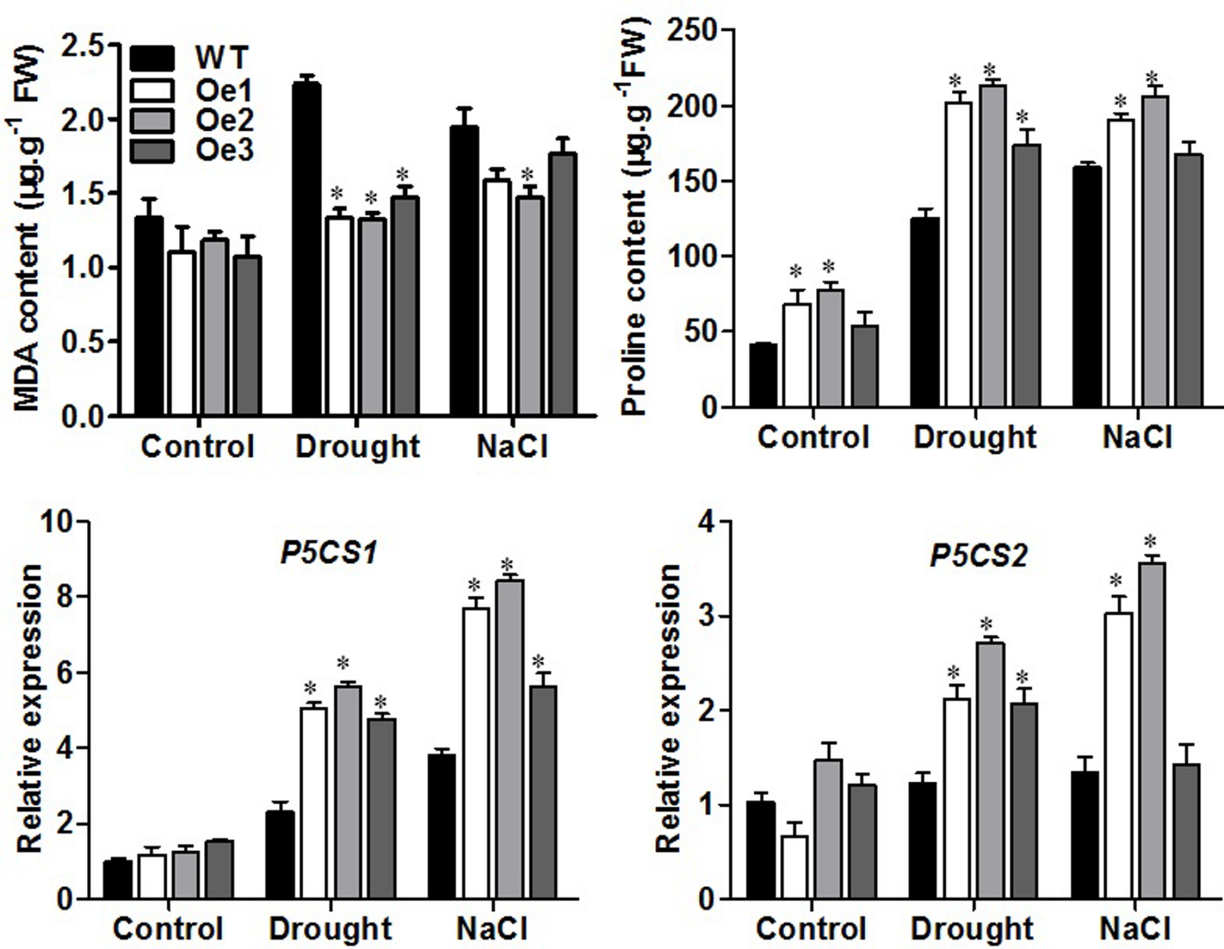
Contents of MDA and proline and analysis of *P5CS1* and *P5CS2* gene expression in WT and transgenic *Arabidopsis* plants with drought or salt stress. Data are means of four biological replicates with error bars indicating SD. Asterisks indicate a significant difference between the WT and the transgenic lines (*P<0.01).

Malondialdehyde (MDA) is used to evaluate ROS-mediated injuries in plants, and proline is an antioxidant under water stress conditions. We measured the contents of MDA and proline, and investigated the expression of the *Arabidopsis* proline biosynthesis genes, 11-Pyrroline-5-Carboxylate Synthase 1 and 2 (*P5CS1* and *P5CS2*) in WT and *ZmPIP1;1*-Oe transgenic plants. In normal growth conditions, contents of MDA and proline had no difference between transgenic lines and the WT (Fig 7). After drought or salt treatment, contents of MDA was significantly lower in *ZmPIP1;1*-Oe transgenic plants than WT (Fig 7), content of proline was increased in *ZmPIP1;1*-Oe transgenic plants than WT did under drought or salt stress. qRT-PCR analysis revealed that gene expression levels of *P5CS1* and *P5CS2* were higher in *ZmPIP1;1*-Oe transgenic plants than in WT under drought and salt stress (Fig 7A and 7B). These results indicated that superior biochemical capabilities of the transgenic plants under drought or salt stress.

## Discussion

Although 31 AQP proteins have been identified in maize genome [13], little is known about the function of AQPs. In our study, ZmPIP1;1 shared a high degree of sequence similarity with many other AQP genes confer to water stress tolerance (S1 Fig) [28–31,42–44]. Amino acid sequence analysis showed that ZmPIP1;1 contains six putative transmembrane a-helices and a conserved sequence which is found in all PIP members. Subcellular localization experiments indicated that the ZmPIP1;1 protein was localized on the plasma membrane and internal membrane compartment endoplasmic reticulum (Fig 1). Previous studies reported that NtAQP1 [45]and PhPIP2;1 [46]were also localized on some endomembrane in protoplasts, which were consistent with our result. Therefore, *ZmPIP1;1* may be involved in regulation of transmembrane transport, which regulates the water uptake and movement in plant. In fact, overexpression of aquaporin in plants has shown either positive or negative effects on stress tolerance [47]. In resent years, several studies have demonstrated the importance of AQP genes in drought and salt stress tolerance [28–31,42,43]. For instance, the overexpression of a banana AQP gene *MusaPIP1;2* improved drought, salt and cold stress tolerance in transgenic plants [30]. Also, Zhou have reported that overexpression of a endogenous gene *GmPIP1;6* in transgenic soybean enhanced salt tolerance and improved the seeds size [31]. But in some cases, overexpression of aquaporin has resulted in sensitivity to water stress [44,48]. For example, overexpression of a barley AQP gene *HvPIP2;1* raised salt sensitivity in rice [48]. And a *Arabidopsis* AQP gene *AtPIP1;2* overexpressed in tobacco has caused reduced drought stress tolerance [44]. Even these PIPs share high sequence homology, the difference pattern of gene expression might indicate that the divergent functions of PIPs in different plants may be attributed to stress antagonism.

In maize, *ZmPIP1;1* was the highest expressed PIPs (S2 Fig) and was high abundance in tassel, ear, leaf, stem and root whereas it was lowly expressed in embryo and endosperm (Fig 2A).These results are similar with previous studies on an AQP gene, *ZmTIP1*, in maize [49]. *ZmTIP1* was highly expressed in maize tassel, ear, embryo, leaves and root, especial in zones of cell division and elongation. However, *ZmTIP1* was absent from the endosperm and seed. This specificity may be due to the highly specialized growth processes observed in these organs. Plant reproduction requires highly controlled cell expansion which needs specialized cell water transport.

In our study, *ZmPIP1;1* was induced after treat with 10% PEG and 100 mM NaCl both in maize root and shoot in different time (Fig 2B and 2C), we supposed that *ZmPIP1;1* may take apart in maize osmotic and salt tolerance. Therefore, to understand the function of *ZmPIP1;1* under drought and salt stress, we generated *ZmPIP1;1* overexpression transgenic *Arabidopsis* plants under the control of the constitutive CaMV 35S promoter (S3 Fig). Two transgenic lines Oe1 and Oe2 showed more higher expression levels of *ZmPIP1;1* than Oe3 in leaves, all three lines were selected for further experiments (Fig 3C). Coincide with the phenotypes of *ZmPIP1;1*-Oe plants improved tolerance to osmotic and salt stress in root when compared with WT (Fig 3A and 3B). These results are consistent with previous studies on AQP genes conferring osmotic and salt stress tolerance in transgenic plants. We further tested the growth of WT and *ZmPIP1;1*-Oe plants, under drought treatment in soil culture. *ZmPIP1;1*-Oe plants did not wilt as severely as WT and recover after re-watered, and showed a significantly higher survival rate than WT (Fig 4A and 4B). These results suggested that *ZmPIP1;1* was required for tolerance to drought stress. This might result from a lower water loss in shoots in *ZmPIP1;1*-Oe plants than did WT under drought stress (Fig 4C). After salt treatment, both WT and *ZmPIP1;1*-Oe plants showed chlorosis, however, the shoot fresh weight of *ZmPIP1;1*-Oe plants were significantly higher compare to WT (S4 Fig). All these results suggested that overexpression of *ZmPIP1;1* improved plant tolerance to drought and salt stress.

Gene expression analysis revealed that ABA-induced gene *RD29A* and *RD29B*, osmotic regulation gene *LEA18*, oxidative-induced gene *TRX5* were responsive to drought or salt stress (Fig 5). In normal growth condition, we observed no significant difference in the expression of tested genes in the transgenic plants compared to WT plants. However, under drought or salt stress condition, the stress responsive genes was significantly higher in the transgenic plants than WT plants, in accord with the phenotypes of *ZmPIP1;1*-Oe transgenic plants in response to these stress. Previous studies reported that the transcriptional levels of stress responsive genes were changed in certain aquaporin gene overexpression transgenic plants that conferred to abiotic stress tolerance [50–52]. *ZmPIP1;1* is rapidly induced by drought and salt stress, and it regulates a number of genes involved in stress response, the results indicating a role of *ZmPIP1;1* in the modulation of osmoprotection. This was further physiologically demonstrated, *ZmPIP1;1*-Oe plants displayed lower levels of stress-induced ROS such as O_2_^-^ and H_2_O_2_ and higher activity of ROS scavenging enzymes such as CAT and SOD under drought and salt stress (Fig 6). ROS are generated in plants under oxidative stress which can cause severe oxidative damage at the tissue and cellular levels. Also, our result showed decreased content of MDA, increased content of proline and higher induction of key proline biosynthetic *P5CS* genes expression in *ZmPIP1;1*-Oe plants compare to WT under drought and salt stress (Fig 7). MDA is used to evaluate ROS-mediated injuries in plants. Proline is an osmoprotectant under water stress conditions, the higher level of proline displays enhanced tolerance to water stress. These results displayed enhanced stress tolerance in *ZmPIP1;1*-Oe transgenic plants, and this might result from increased activities of ROS scavenging enzymes and enhanced expression of proline biosynthetic genes under drought and salt stress. Thus, induced expression of stress responsive genes and increased activities of ROS scavenging enzymes suggested that *ZmPIP1;1* regulated abiotic responses share a common molecular mechanism during the specific signal transduction, especially in the modulation of cellular osmoprotection and cellular redox and ROS homeostasis. Overexpression of *ZmPIP1;1* might strengthen the sense to osmotic stress signals and lead to less damage caused to the cell membrane which could protect the protein machinery from dehydration damage and eventually induce enhanced stress defense. This widened our understanding on the coordination of plant responses to different abiotic stress. Previous reports proposed that aquaporins represent one of the earliest celluar responses to stresses and might possibly be extended to other membrane proteins. In addition, the dynamic responses of aquaporins to osmotic stress supposed that aquaporins which localized on the plasma membrane may serve as osmosensors [14,47]. Further studies should be required to emphasize the mechanism of PIPs involved in abiotic stress tolerance.

In conclusion, we found that *ZmPIP1;1* is rapidly induced upon water stress, and it can improve tolerance to drought and salt stress when overexpressed in *Arabidopsis* not only by up-regulation of stress responsive genes which involved in cellular osmoprotection and oxidative detoxification but also by increasing of ROS scavenging enzymes activities. Further studies should be conducted to understand the mechanism and function of *ZmPIP1;1* for crop improvement under abiotic stress.

## Supporting information

S1 Fig Comparison of the amino acid sequences of PIPs. Phylogenetic analysis of ZmPIPs and other PIPs.
(TIF)

S2 Fig Expression pattern of *ZmPIP1;1* under normal condition. Relative expression levels of *ZmPIP1;1* in leaf and root. Trifoliolate maize seedlings were grown in nutrient solution. Total RNA was extracted from different tissues for qRT-PCR. All data are means of three biological replicates with error bars indicating SD.
(TIF)

S3 Fig Schematic illustration of T-DNA sequence of *ZmPIP1;1* overexpression vector. (TIF)

S4 Fig Phenotypes of *ZmPIP1;1* overexpression transgenic lines under salt treatment. (A) Growth of WT and *ZmPIP1;1*-Oe transgenic *Arabidopsis* seedlings under normal and salt conditions for 7 days. (B) Shoot fresh weight of different genotypes under normal and salt conditions. Three independent repeats were performed, each data are means of 5 plants with error bars indicating SD, * P<0.01.
(TIF)

S1 Table Primers used in this study. (XLSX)
