# Correction: Overexpression of a maize plasma membrane intrinsic protein ZmPIP1;1 confers drought and salt tolerance in *Arabidopsis*

**DOI:** 10.1371/journal.pone.0218234

**Published:** 2019-06-06

**Authors:** Lian Zhou, Jing Zhou, Yuhan Xiong, Chaoxian Liu, Jiuguang Wang, Guoqiang Wang, Yilin Cai

After publication of this article [1], concerns were raised regarding the following points:

1. Relevant literature on ZmPIP1;1 may not have been cited and discussed in the manuscript, including previous studies on the expression of this isoform in different tissues and under different conditions.
2. PEG-induced osmotic stress has been referred to as ‘drought’ stress, whereas PEG-induced stress and in-soil drought stress are not directly physiologically comparable.
3. The limitations of antioxidant activities as indicators of stress intensity are not clearly articulated.

With respect to point (1), the authors acknowledge that a reference to a previous study [2] should have been included in order to provide context for their study and its results. The authors note that ZmPIP1;1 showed the highest level of expression of all PIP isoforms in both [1] and [2].

In terms of point (2), the authors acknowledge that PEG-induced stress and in-soil drought stress are not directly comparable, and this distinction should have been clearly articulated in the text. Similarly, with respect to point (3), the authors recognise that antioxidant activities alone are not commonly considered to represent comprehensive indicators of stress intensity. This too should have been noted in the text of the article.
