# Supplementary figures and images for: Overexpression of a maize plasma membrane intrinsic protein ZmPIP1;1 confers drought and salt tolerance in Arabidopsis
Source: PLoS One. 2018 Jun 1;13(6):e0198639. doi: 10.1371/journal.pone.0198639 (PMC5983466; doi:10.1371/journal.pone.0198639)

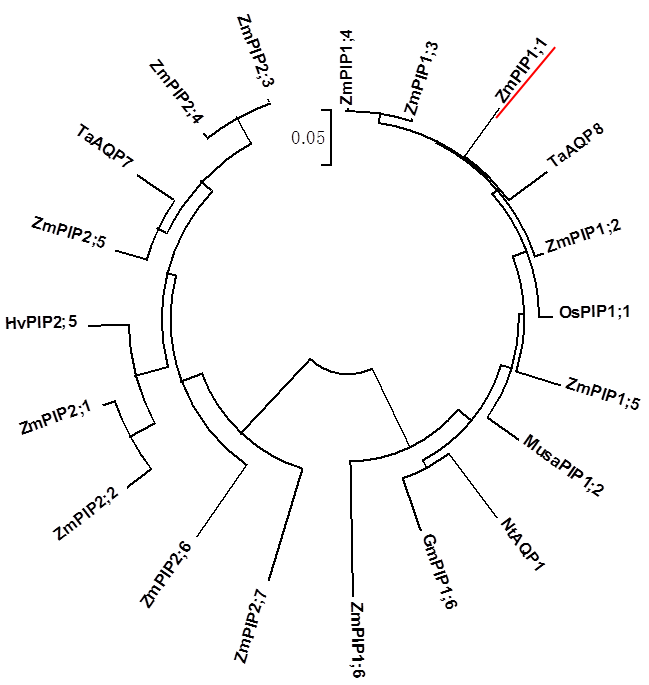

Supplement: S1 Fig — Phylogenetic analysis of ZmPIPs and other PIPs. (TIF) [file pone.0198639.s001.tif]

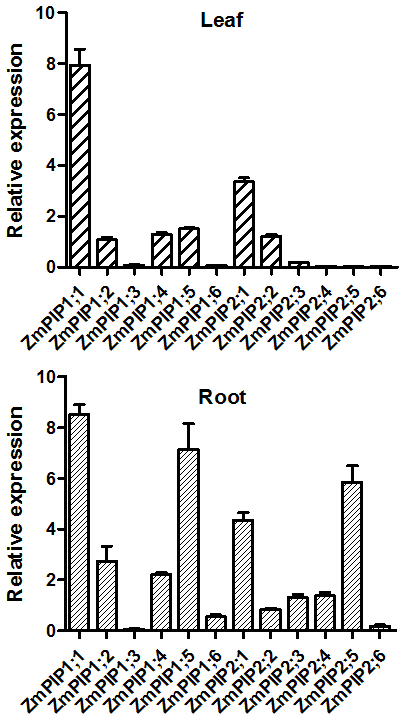

Supplement: S2 Fig — Relative expression levels of ZmPIP1;1 in leaf and root. Trifoliolate maize seedlings were grown in nutrient solution. Total RNA was extracted from different tissues for qRT-PCR. All data are means of three biological replicates with error bars indicating SD. (TIF) [file pone.0198639.s002.tif]

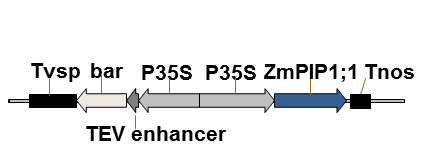

Supplement: S3 Fig — (TIF) [file pone.0198639.s003.tif]

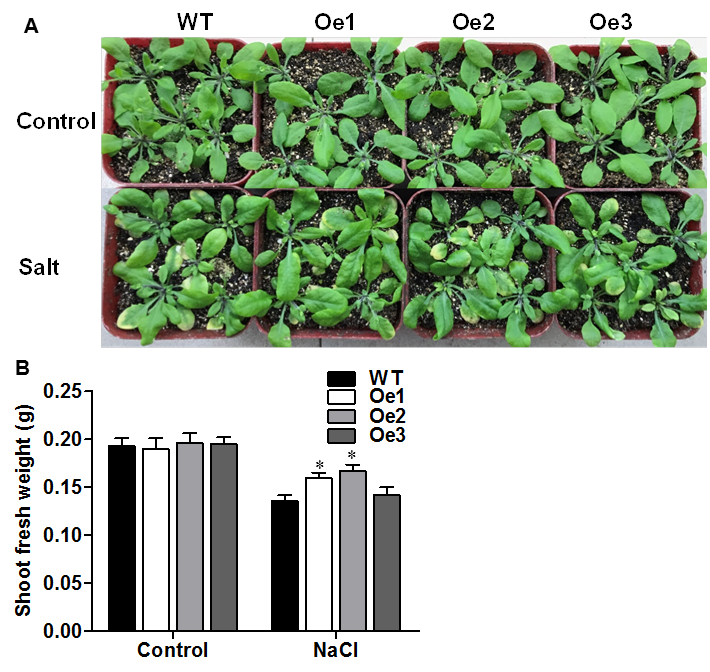

Supplement: S4 Fig — (A) Growth of WT and ZmPIP1;1-Oe transgenic Arabidopsis seedlings under normal and salt conditions for 7 days. (B) Shoot fresh weight of different genotypes under normal and salt conditions. Three independent repeats were performed, each data are means of 5 plants with error bars indicating SD, * P<0.01. (TIF) [file pone.0198639.s004.tif]
